# Racial/Ethnic Disparities in VA Services Utilization as a Partial Pathway to Mortality Differentials among Veterans Diagnosed with TBI

**DOI:** 10.5539/gjhs.v8n2p260

**Published:** 2015-07-20

**Authors:** Clara E. Dismuke, Mulugeta Gebregziabher, Leonard E. Egede

**Affiliations:** 1 Health Equity and Rural Outreach Innovation Center, Ralph H. Johnson Veterans Affairs Medical Center, Charleston, SC, USA; 2 Center for Health Disparities Research and Division of General Internal Medicine & Geriatrics, Medical University of South Carolina, Charleston, SC, USA; 3 Department of Public Health Sciences, Medical University of South Carolina, Charleston, SC, USA

**Keywords:** traumatic brain injury (TBI), veterans, access/equity, racial/ethnic disparities, utilization, mortality, mediation, Cox

## Abstract

**Objective:**

Primary: To examine Veterans Administration (VA) utilization and other potential mediators between racial/ethnic differentials and mortality in veterans diagnosed with traumatic brain injury (TBI).

**Design:**

A national cohort of veterans clinically diagnosed with TBI in 2006 was followed from January 1, 2006 through December 31, 2009 or until date of death. Utilization was tracked for 12 months. Differences in survival and potential mediators by race were examined via K-Wallis and chi-square tests. Potential mediation of utilization in the association between mortality and race/ethnicity was studied by fitting Cox models with and without adjustment for demographics and co-morbidities. Poisson regression was used to study the association of race/ethnicity with utilization of specialty services potentially important in the management of TBI.

**Setting:**

United States (US) Veterans Administration (VA) Hospitals and Clinics.

**Participants:**

14, 690 US veterans clinically diagnosed with TBI in 2006.

**Interventions:**

Not Applicable. The study is a secondary data analysis.

**Main Outcome Measures:**

Mortality, Utilization.

**Results:**

Hispanic veterans were found to have significantly higher unadjusted mortality (6.69%) than Non-Hispanic White veterans (2.93%). Hispanic veterans relative to Non-Hispanic White were found to have significantly lower utilization of all services examined, except imaging. Neurology was found to be the utilization mediator with the highest percent of excess risk (3.40%) while age was the non utilization confounder with the highest percent of excess risk (31.49%). In fully adjusted models for demographics and co-morbidities, Hispanic veterans relative to Non-Hispanic Whites were found to have less total visits (IRR 0.89), TBI clinic (IRR 0.43), neurology (IRR 0.35), rehabilitation (IRR 0.37), and other visits (IRR 0.85) with only higher mental health visits (IRR 1.53).

**Conclusions:**

We found evidence that utilization is a partial mediator between race/ethnicity and mortality, especially neurology utilization. We also found that Hispanic veterans receive significantly less TBI clinic, neurology, rehabilitation and other types of utilization. The use of innovative system factors (decision aids, information tools, patient activation, and adherence support interventions) could be valuable in enhancing utilization of specific TBI related services, especially among ethnic minorities.

## 1. Introduction

Striving to be an equal access system for veterans of all race/ethnicity, the Veterans Affairs (VA) health care system has been referred to as a “beacon of hope” for a potential health reform model in the US (Marmor, 2007). Even so, there is recent evidence of racial/ethnic disparities in mortality among veterans diagnosed with traumatic brain injury (TBI), one of the “signature” injuries from the conflicts in Iraq and Afghanistan (Egede, Dismuke, Echoles, 2012). Disparities in health care access for other disorders have also been found in the VA system of care. Studies have shown that African American and Latino veterans are less likely to access joint replacement surgery and analgesic medication in osteoarthritis and pain management than White veterans (Oddone, Horner, Monger, & Matchar, 1993; Oddone et al., 1999; Oddone et al., 2002). African American veterans are less likely to access potentially curative surgical resection, yet equally likely to access non-surgical interventions (Sedlis, Fisher, Tice, Esposito, Madmon, & Steinberg, 1997; Maynard, Sun, Lowy, Sales & Fihn, 2006; Conigliaro, et al., 2000; Mickelson, Blum, & Geraci, 1997). There have been mixed findings regarding racial disparities in the use of invasive procedures in veterans with cardiovascular disease, but most studies have found that non-White veterans access fewer procedures than White veterans (Goldstein, Matchar, Hoff-Lindquist, Samsa, & Horner, 2003; Peterson, Wright, Daley, & Thibault, 1994; Whittle, Congliaro, Good, & Lofgren, 1993; Conigliaro et al.,2000; Petersen, Wright, Petersen, & Daley, 2002; Kressin et al., 2004; Groeneveld, Kruse, Chen, & Asch, 2007; Collins, Johnson, Henderson, Khuri, & Daley, 2002). There have also been mixed findings with regard to preventive care where no racial disparities have been found in colorectal cancer screening and lipid lowering therapy (Dolan et al., 2005; Fisher, Jeffreys, Coffman, & Fasanella, 2006; Etzioni et al., 2006; Woodward, Kressin, & Petersen, 2004), yet African American veterans have been found to have less access to influenza vaccines (Straits-Troster, Kahwati, Kinsinger, Orellen, Burdick, & Yevich, 2006).

Racial/ethnic disparities have also been found in the civilian literature in hospitalization, emergency department (ED) and rehabilitation services for individuals with TBI. African American females have been found to be less likely to be hospitalized than white females (Selassie, Pickelsimer, Fraizer, & Ferguson, 2004). American Indian/Alaska Natives have been found to have the highest hospitalization rates while Asian/Pacific Islanders have the lowest rates (Rutland-Brown, Wallace, Faul, & Langlois, 2005). However, children have not been found to have significant disparities in hospitalization by race/ethnicity (Howard, Joseph, & Natale, 2005). Racial/ethnic disparities have also been found in the civilian literature for Emergency Department (ED) services. African Americans have been found to be more likely to receive ED care from a resident physician than from a staff physician and less likely to be sent back to a referring physician after discharge (Bazarian, Pope, McClung, Cheng, & Flesher, 2003). Hispanics have been found to be 6 times more likely to receive a nasogastric tube than non-Hispanics (Bazarian, Pope, McClung, Cheng, & Flesher, 2003). However, an alternative study found no significant racial/ethnic differences in ED care (Shafi & Gentilello, 2000).

Racial/ethnic disparities have also been found in civilian rehabilitation services for TBI. Post- hospitalization, African American and Hispanics have been found in several studies to be significantly less likely to be placed in rehabilitation than Whites (Shafi, et al.,2007; Bowman, Martin, Sharar, & Zimmerman, 2007; Chang, Ostir, Kuo, Granger, & Ottenbacher, 2008). Even so, one study among children with TBI found African American children were more likely to be placed in inpatient rehabilitation,^31^ and another found no significant differences by race/ethnicity in rehabilitation of individuals with TBI.^32^ An additional study found that minorities received less physical, occupational, speech-language and psychotherapy than Whites (Burnett, 2003). Racial disparities can be due to a number of reasons based on a published conceptual model that include environmental factors (concentrations of poverty, proximity to services, lack of information and education, poor housing conditions and environment), individual (health behaviors, social norms, social support, stress & psychosocial), access to specialty and other specific services, medical practice patterns, patient-provider cultural concordance, access to high quality hospitals (Sarrazin, Campbell, Richardson, & Rosenthal, 2009).

There is good evidence for racial/ethnic disparities in mortality of veterans with TBI and racial/ethnic disparities in access to health care services by civilians with TBI. The conceptual reasons for this disparity may come from several sources among antecedent (race/ethnicity, gender, age, insurance, marital status, urban/rural) and or system factors (region of VA facility, patient comorbidites), resulting in process (utilization) and consequently clinical outcome (mortality) disparities (Coyle & Bates, 1999) Given the absence of studies on the association of race/ethnicity with utilization of VA services, we sought to test a primary hypothesis that disparities in a process measure, utilization, would be at least a partial pathway (mediator) for the significant antecedent (racial/ethnic) differential in a clinical outcome (mortality) which has been found among veterans with TBI (Marmor, 2007).

## 2. Methods

### 2.1 Subjects

We identified 14,690 veterans who were seen in VA medical centers and community based outpatient clinics between January 1, 2006 and December 31, 2006 with clinically diagnosed TBI (diagnosed at any time) using definitions established in a prior VA study which included all ICD-9 codes used by the Center for Disease Control (CDC) as well as ICD-9 codes for postconcussive syndrome and TBI-related late effects mandated by the VA (Carlson, Nelson, Orazem, Nugent, Cifu, & Sayer, 2010).

### 2.2 Cohort Creation and Variables

A national cohort of veterans with TBI was created by linking multiple patient and administrative files from 2 large VA databases. These two data bases, namely the Veterans Health Administration (VHA) Decision Support System (DSS) and Vital Status files were linked by scrambled Social Security Number (SSN). The VHA DSS is a national automated management information system based on commercial software to integrate data from clinical and financial systems for both inpatient and outpatient care. Veterans with clinically diagnosed TBI were identified based on having an ICD-9 code for TBI (according to VA coding guidelines with a primary diagnosis of 800.xx, 801.xx, 803.xx, 804.xx, 851.xx, 852.xx, 853.xx, 854.xx or V57.xx along with a TBI secondary diagnosis code of 800.x, 801.xx, 803.xx, 804.xx, 851.xx, 852.xx, 853.xx, 854.xx, 310.2, 905.0 or 907.0) (Carlson, Nelson, Orazem, Nugent, Cifu, & Sayer, 2010) in the patient treatment file (PTF). We utilize the term clinically diagnosed TBI to distinguish from the VA primary care TBI screen which was implemented in the VA for all Operation Enduring Freedom/Operation Iraqi Freedom/Operation New Dawn (OEF/OIF/OND) veterans since September of 2007. A positive result on the primary screen is only an indicator of possible TBI. A secondary exam by a specialist is required to confirm TBI. We only include veterans with confirmed TBI in this study. We used these criteria to assemble a retrospective cohort of 14,690 veterans who had an ICD-9 code for clinically diagnosed TBI between January 1, 2006 and December 31, 2006.

Utilization, socio-demographic and clinical variables were collected from the PTF file. Date of death was obtained from the Vital Status file between January 1, 2006 and December 31, 2009. The main outcome measure was time to death which was defined as months to death between date of entry into the study in 2006 and date of death or December 31, 2009. Type of service was based on clinic stop codes taken from the FY2006 VIReC Research User Guide (RUG): TBI clinic (219); neurology (315); imaging (150,151); rehabilitation (202-208, 213, 214, 216, 217, 220, 222, 228, 229); mental health and substance abuse (502, 503, 505, 506, 509, 510, 512-516, 519, 522-525, 527-533, 535-538, 540, 542, 545-547, 550, 552, 553, 554, 557-570, 573-581, 589). All other services not included in these categories were grouped together.

Race/ethnicity was based on self-report and classified as non-Hispanic White (NHW), non-Hispanic Black (NHB), Hispanic, and other/missing due to the number of veterans with missing race. The race and ethnicity of the patient was taken from the Veterans Administration 2006 Outpatient Medical SAS data files. Currently, race can be categorized by using the following variables: race (race or national origin) or race1-race7 (race with collection methods). Using the race variable alone produced a data file with approximately 58% of race identified as other or missing. An algorithm was created using both race and race1-race6 variables to decrease this number to approximately 32%.

Other risk factors (covariates) included age, marital status, gender, urban residence, VA region of residence, insurance status and ICD-9 coded comorbidities (cerebrovascular disease, depression, lung disease, substance abuse, anemia, cancer, congestive heart failure, cardiovascular disease, electrolyte disorders, hypertension hypothyroidism, liver disease, psychoses, obesity peripheral vascular disease, other diseases) using previously validated algorithms (Quan et al., 2005).

### 2.3 Statistical Analysis

We performed three types of analyses. First, we tested for unadjusted racial/ethnic differences in survival using Kruskal Wallis, and mortality and potential mediators by racial category using chi-square. Second, in order to examine potential mediators between race/ethnicity and mortality we followed a method specific to Cox models (Park, Buist, Tiro, & Taplin, 2008; Barron & Kenny, 1986). First we estimated a Cox model for race alone to ascertain the hazard ratios on race. Second separate Cox models including race and each potential mediator were estimated to ascertain the mediator adjusted hazard ratios on race. Third, excess risk percentage (amount of mortality explained by the potential mediator in presence of race) was calculated as: [(hazard ratio on race in race only model – hazard ratio on race in race + potential mediator model)/hazard ratio on race in race only model)] *100. Fourth, a model adjusting for all potential mediators was estimated. Time to death was defined as the number of months from time of entry into the study in 2006 until time of death or censoring (December 31, 2009). Death was coded as 1 if death occurred and 0 if censored. We dealt with tied event times in the Cox survival models using Breslow and Efron methods that provided approximations to the exact conditional probability. We used the robust sandwich estimate of Lin and Wei (1989) for the covariance matrix to check the robustness of the model based covariance matrix estimate. Finally, we performed graphical and numerical methods of Lin, Wei, and Ying (1993) for checking the adequacy of the Cox regression model. Finally, to examine the association of race with total and specialty service utilization potentially important to TBI management, we estimated Poisson models for all services, TBI clinic, imaging, neurology, rehabilitation, mental health/substance abuse and all other services. All data analyses were conducted using STATA Version 9.0 (Stata Corp, 2005). This study was approved by the Medical University of South Carolina Institutional Review Board and the Ralph H. Johnson Veterans Administration Research & Development Committee.

## 3. Results

### 3.1 Unadjusted Analyses

Table 1 shows mortality, survival time, and utilization measures by racial/ethnic group. Mortality rates were significantly different by race with 6.7% of Hispanic veterans with clinically diagnosed TBI having died relative to 2.9% of NHW veterans during the study period. Survival time was also significantly different by race with a mean survival time of 45.17 months for Hispanic relative to 46.23 months for NHW veterans. Hispanic veterans relative to NHW were found to have significantly lower unadjusted utilization of Total visits (1.96 vs. 2.46), TBI clinic visits (0.02 vs. 0.89), neurology visits (0.04 vs. 0.17), rehabilitation (0.08 vs. 0.19), mental health/substance abuse (0.62 vs. 0.66), and other types of visits (1.17 vs. 1.33).

**Table 1:** Differences in proportions and means of model outcomes and covariates by race (N=14,690)

	Non-Hispanic White n=7,885	Non-Hispanic Black n=1,748	Hispanic n=314	Other/Missing Race n=4,743
**Outcomes**				
Died*	2.93%	2.69%	6.69%	1.37%
Survival Months*	46.23	46.25	45.17	46.62
Total_Visits*	2.46	2.73	1.96	2.65
TBI Clinic Visits	0.89	0.07	0.02	0.17
Neurology*	0.17	0.19	0.04	0.14
Imaging	0.02	0.01	0.02	0.02
Rehabilitation*	0.19	0.32	0.08	0.44
Mental Health/Substance Abuse*	0.66	0.77	0.62	0.58
Other Visits*	1.33	1.36	1.17	1.29
**Covariates**				
Male*	94.08%	92.91%	98.73%	90.81%
Age*				
Under Age 50	31.68%	39.53%	18.79%	
Age 50-64	43.06%	46.00%	36.94%	
Age 65-74	11.17%	7.32%	18.15%	
Age 75+	14.09%	7.15%	26.11%	
VA Only Insurance*	55.29%	70.14%	59.55%	65.91%
Medicare*	31.29%	18.48%	33.76%	19.99%
Private Insurance*	13.42%	11.38%	6.69%	14.10%
Married*	41.45%	28.95%	51.91%	41.03%
Urban*	62.50%	85.58%	90.76%	66.43%
Region*				
Northeastern	12.75%	10.93%	4.78%	9.49%
Mid-Atlantic	19.57%	27.86%	1.27%	16.70%
Southern	23.40%	31.98%	62.74%	23.42%
Midwestern	25.29%	18.99%	10.83%	21.95%
Western	18.92%	10.24%	20.38%	28.25%
Cerebrovascular* Disease	11.64%	11.04%	5.7%	9.2%
Depression*	6.34%	4.41%	3.50%	6.81%
Lung Disease*	2.82%	1.66%	1.27%	2.30%
Substance Abuse*	6.01%	9.84%	4.46%	5.36%
Anemia	0.88%	1.09%	0.32%	0.70%
Cancer	1.19%	0.80%	0.64%	0.84%
Congestive Heart Failure	0.47%	0.44%	0.63%	0.46%
Cardiovascular Disease	0.27%	0.00%	0.63%	0.25%
Electrolyte Disorders	0.00%	0.00%	0.64%	0.44%
Hypertension*	11.29%	11.56%	6.69%	11.95%
Hypothyroidism*	1.27%	0.40%	0.32%	1.27%
Liver Disease	1.10%	0.97%	0.32%	0.93%
Psychoses*	5.19%	6.41%	1.91%	4.26%
Obesity*	2.48%	1.60%	0.64%	2.1%
Peripheral Vascular Disease	0.62%	1.31%	0.11%	0.48%
Other Diseases	0.73%	1.31%	0.32%	0.86%

* indicates significant at P<0.05.

### 3.2 Adjusted Analyses

#### 3.2.1 Main Outcome

Table 2 shows the hazard ratios and percentage excess risk (ER) from the potential utilization and other mediators. The hazard ratio on Hispanic in the race only model was 2.35 (95% CI 1:50:3.68). Significant utilization mediators were total visits (HR 0.93 95% CI 0.88:098 ER 2.12%), imaging (HR 1.91 95% CI 1.19:3.04 ER -0.42%), mental health/substance abuse (HR 0.81 95% CI 0.69:0.93 ER 1.70%), and other visits (HR 0.99 95% CI 0.96:1.03 ER 0.00%) with the strongest being neurology (HR 0.68 95% CI 0.50:0.91). Significant non-utilization mediators were male (ER 4.25%), insurance (2.55%), married (3.83%), co-morbidities (3.40%) with the highest excessive risk percentage being age (31.49%). In a fully adjusted model for all potential utilization and other mediators, the hazard ratio on Hispanic becomes non significant.

**Table 2:** Hazard ratios and % excess risk^a^

	Race Only Hazard Ratios (95% CI)	Potential Mediator Hazard Ratios^b^ (95% CI)	% Excess Risk^a^	Race + All Potential Mediators Hazard Ratios (95% CI)
Hispanic	2.35*(1.50:3.68)			1.58(0.98:2.54)
Non Hispanic Black	0.93(0.68:1.27)			1.24(0.90:1.72)
Other/Unknown Race	0.45*(0.34:0.60)			0.58*(0.44:0.77)
Total Visits		0.93*(0.88:0.98)	2.12%	
TBI Clinic		0.62(0.33:1.16)	0.85%	0.82(0.48:1.39)
Neurology		0.68*(0.50:0.91)	3.40%	0.81(0.60:1.09)
Imaging		1.91*(1.19:3.04)	-0.42%	1.12(0.68:1.84)
Rehabilitation		0.81(0.63:1.05)	0.85%	0.93(0.74:1.15)
Mental Health/Substance Abuse		0.81*(0.69:0.93)	1.70%	0.93(0.83:1.04)
Other Visits		0.99(0.96:1.03)	0.00%	1.00(0.97:1.03)
Male		8.24*(2.64:25.69)	4.25%	5.39*(1.72:16.86)
Age			31.49%	
Age 50-64		4.58*(2.85:7.38)		4.10*(2.54:6.62)
Age 65-74		12.31*(7.49:20.22)		8.37*(4.96:14.11)
Age 75+		22.86*(14.32:36.49)		14.67*(8.87:24.25)
Insurance			2.55%	
Medicare		3.93*(3.13:4.93)		1.62*(1.24:2.11)
Private Insurance		1.16(0.78:1.74)		1.04(0.69:1.56)
Married		1.43*(1.16:1.76)	3.83%	0.96(0.77:1.19)
Urban		1.07(0.85:1.34)	2.13%	1.10(0.87:1.40)
Region			-1.28%	
Mid-Atlantic		1.10(0.76:1.60)		1.25(0.86:1.82)
Southern		0.97(0.67:1.40)		1.01(0.70:1.46)
Midwestern		0.88(0.61:1.28)		1.08(0.74:1.57)
Western		0.92(0.61:1.33)		1.01(0.68:1.49)
Co-morbidities			3.40%	
Cerebrovascular Disease		0.61*(0.41:0.92)		0.70(0.46:1.06)
Depression		0.35*(0.17:0.72)		0.51(0.25:1.03)
Lung Disease		2.06*(1.27:3.33)		1.91*(1.18:3.08)
Substance Abuse		0.70(0.42:1.17)		1.08((0.64:1.81)
Anemia		1.52(0.67:3.49)		1.26(0.55:2.88)
Cancer		1.56(0.73:3.34)		0.90(0.42:1.95)
Congestive Heart Failure		3.47*(1.50:7.99)		2.14(0.93:4.92)
Cardiovascular Disease		2.63(0.84:8.30)		1.75(0.55:5.58)
Electrolytes		1.39(0.34:5.59)		0.80(0.19:3.25)
Hypertension		1.05(0.76:1.46)		0.76(0.54:1.05)
Hypothyroidism		1.02(0.41:2.54)		0.87(0.35:2.16)
Liver Disease		1.78(0.79:4.04)		1.72(0.75:3.93)
Psychosis		0.89(0.54:1.48)		1.14(0.68:1.92)
Obesity		0.44(0.16:1.19)		0.62(0.23:1.68)
Peripheral Vascular Disease		0.80(0.20:3.27)		0.53(0.13:2.16)
Other Disease		2.81*(1.44:5.52)		2.01*(1.02:3.99)

* indicates significant at P<0.05.

a [(Hazard Ratio on Race in Race only model – Hazard Ratio on Race in Race + Potential Mediator Model)/ Hazard Ratio on Race in Race only model] * 100.

b Model includes race + each potential mediator.

#### 3.2.2 Secondary Outcome

**Specialty Service Utilization**

Table 3 contains the incidence rate ratios (IRR) of total, TBI clinic, neurology, imaging, mental health, rehabilitation, and other services estimated using Poisson regressions. Hispanic ethnicity was found to be statistically significantly associated with 11% lower (0.89; 95% CI 0.82:0.97) total visits, 57% lower (0.43 95% CI: 0.21:0.86) TBI clinic, 65% lower neurology (0.35; 95% CI 0.21:0.60), 63% lower (0.37; 95% CI 0.25:0.56) rehabilitation and 15% lower other visits (0.85; 95% CI 0.77:0.95). Only in mental health was Hispanic ethnicity statistically significantly associated with 53% higher utilization (1.53; 95% CI 1.32:1.77) relative to NHW.

**Table 3:** Incidence rate ratios from poisson count models of total and specialty utilization (95% confidence intervals)

	Total Visits	TBI Clinic	Neurology	Imaging	Rehab	Mental Health	Other Visits
Hispanic	0.89* (0.82:0.97)	0.43* (0.21:0.86)	0.35* (0.21:0.60)	0.72 (0.29:1.79)	0.37* (0.25:0.56)	1.53* (1.32:1.77)	0.85* (0.77:0.95)
Non Hispanic Black	1.03 (0.99:1.06)	0.99 (0.81:1.20)	1.07 (0.95:1.22)	0.88 (0.57:1.34)	1.42* (1.29:1.57)	1.00 (0.94:1.06)	0.99 (0.95:1.04)
Other/Unknown Race	1.05* (1.03:1.08)	1.76* (1.59:1.95)	0.89* (0.81:0.98)	1.33* (1.04:1.69)	1.92* (1.79:0.2.05)	0.93 (0.89:0.98)	0.95* (0.92:0.98)
Male	1.38* (1.32:1.44)	2.89* (2.23:3.74)	1.04 (0.88:1.22)	0.78 (0.52:1.17)	1.36* (1.22:1.52)	2.19* (1.97:2.43)	1.13* (1.06:1.19)
Age 50-64	0.81* (0.79:0.82)	0.36* (0.32:0.40)	1.12* (1.02:1.22)	1.02 (0.75:1.39)	0.32* (0.30:0.35)	1.00 (0.95:1.04)	0.92* (0.89:0.95)
Age 65-74	0.62* (0.60:0.65)	0.20* (0.15:0.27)	0.97 (0.82:1.13)	1.45 (0.96:2.21)	0.25* (0.21:0.30)	0.37* (0.33:0.41)	0.88* (0.83:0.93)
Age 75+	0.55* (0.53:0.58)	0.08* (0.05:0.12)	0.57* (0.47:0.69)	2.95* (2.09:4.18)	0.34* (0.29:0.39)	0.15* (0.13:.17)	0.85* (0.80:0.89)
Medicare	1.05* (1.02:1.08)	1.06 (0.91:1.23)	0.84* (0.75:0.95)	2.96* (2.08:4.19)	0.88* (0.80:0.97)	1.17* (1.10:1.23)	1.05* (1.01:1.09)
Private Insurance	1.20* (1.16:1.23)	1.05 (0.91:1.21)	0.99 (0.88:1.12)	8.29* (6.09:11.29)	1.25* (1.14:1.36)	1.71* (1.62:1.80)	0.94* (0.90:0.99)
Married	0.82* (0.81:0.84)	0.82* (0.74:0.92)	1.24* (1.14:1.35)	1.13 (0.89:1.42)	0.67* (0.63:0.72)	0.66* (0.63:0.69)	0.90* (0.88:0.93)
Urban	1.10* (1.07:1.12)	0.68* (0.86:1.38)	1.21* (1.10:1.33)	1.41* (1.09:1.82)	1.19* (1.11:1.27)	1.07* (1.02:1.12)	1.12* (1.08:1.15)
Mid-Atlantic	1.01 (0.97:1.05)	0.46* (0.25:0.87)	0.68* (0.60:0.77)	1.86* (1.23:2.82)	0.86* (0.76:0.99)	1.07* (1.00:1.14)	1.10* (1.04:1.16)
Southern	0.92* (0.89:0.95)	5.13* (3.31:7.94)	0.36* (0.32:0.42)	1.13 (0.73:1.74)	1.84* (1.64:2.07)	0.50* (0.47:0.54)	1.16* (1.10:1.22)
Midwestern	1.11* (1.07:1.15)	24.78* (16.25:37.79)	0.53* (0.46:.0.60)	1.63* (1.08:2.46)	1.43* (1.27:1.61)	0.85* (0.79:0.90)	1.10* (1.05:1.17)
Western	0.87* (0.83:0.90)	3.14* (2.00:4.93)	0.51* (0.45:0.58)	1.06 (0.68:1.66)	1.14* (1.00:1.28)	0.47* (0.43:0.50)	1.18* (1.12:1.25)
Cerebro-vascular Disease	1.01 (0.97:1.04)	0.32* (0.24:0.42)	4.04* (3.70:4.41)	0.41* (0.24:0.71)	1.41* (1.29:1.55)	0.35* (0.31:0.38)	0.79 (0.57:1.09)
Depression	1.10* (1.06:1.15)	0.89 (0.72:1.10)	0.50* (0.40:0.64)	0.25* (0.09:0.69)	0.80* (0.69:0.92)	1.72* (1.61:1.84)	0.99 (0.93:1.05)
Lung Disease	0.97 (0.90:1.04)	0.22* (0.09:0.54)	0.42* (0.26:0.68)	0.37 (0.09:1.53)	0.86 (0.64:1.17)	0.93 (0.78:1.10)	1.06 (0.97:1.16)
Substance Abuse	0.87* (0.84:0.91)	0.25* (0.17:0.37)	0.46* (0.35:0.59)	0.78 (0.38:1.58)	0.25* (0.20:0.32)	1.57* (1.47:1.67)	0.69* (0.64:0.74)
Anemia	1.22* (1.09:1.37)		1.08 (0.65:1.82)	0.61 (0.08:4.43)	0.17* (0.05:0.53)	0.40* (0.26:0.63)	1.61* (1.42:1.82)
Cancer	0.84* (0.74:0.96)		0.49 (0.23:1.05)	0.34 (0.04:2.46)	0.50 (0.25:1.01)	0.36* (0.23:0.57)	1.02 (0.89:1.16)
Congestive Heart Failure	0.81* (0.66:0.98)	0.94 (0.23:3.79)	1.04 (0.49:2.20)	0.75 (0.10:5.45)	0.44 (0.14:1.37)	0.51 (0.25:1.02)	0.85 (0.68:1.06)
Cardio-vascular Disease	0.59* (0.43:0.80)			2.35 (0.69:7.98)		0.11* (0.02:0.47)	0.79 (0.57:1.09)
Electrolytes	0.74* (0.60:0.90)	0.30 (0.07:1.20)	0.56 (0.23:1.37)	0.86 (0.12:6.18)	0.08* (0.01:0.59)	0.15* (0.05:0.40)	1.11 (0.89:1.37)
Hypertension	0.77* (0.75:0.81)	0.09* (0.05:0.16)	0.46* (0.38:0.55)	0.36* (0.20:0.64)	0.21* (0.17:0.27)	0.30* (0.27:0.34)	1.18* (1.13:1.23)
Hypo-thyroidism	0.84* (0.75:0.94)		0.46* (0.25:0.83)		0.08* (0.02:0.77)		1.18* (1.05:1.33)
Liver	0.75* (0.66:0.84)	0.23* (0.05:0.93)	0.46* (0.25:0.86)	0.51 (0.07:3.69)	0.40* (0.20:0.77)	0.32* (0.22:0.46)	1.08 (0.95:1.24)
Psychoses	1.01 (0.97:1.06)	0.43* (0.32:0.59)	0.70* (0.55:0.89)	0.28* (0.10:0.76)	0.14* (0.09:0.20)	2.22* (2.08:2.36)	0.62* (0.57:0.67)
Obesity	0.84* (0.77:0.91)	0.43* (0.23:0.80)	0.52* (0.34:0.80)	0.30 (0.04:2.19)	0.25* (0.15:0.41)	0.88 (0.74:1.03)	0.99 (0.90:1.09)
Peripheral Vascular Disease	1.10 (0.94:1.28)	1.22 (0.39:3.81)	0.63 (0.26:1.52)	0.65 (0.09:4.74)	1.45 (0.84:2.52)	0.31* (0.13:0.69)	1.22* (1.03:1.46)
Other Disease	0.91 (0.80:1.04)	0.18 (0.02:1.33)	0.41* (0.18:0.92)	1.23 (0.36:4.14)	0.13* (0.04:0.41)	0.44* (0.29:0.66)	1.21* (1.05:1.39)

* indicates significant at P<0.05.

Reference groups were: Non-Hispanic White, Under Age 50, VA insurance only, North-Eastern Region, No Co-morbidities. Co-morbidities are in blank when they had missing 95% CI on a first estimate of a model.

## 4. Discussion

A statistically significant difference in race-adjusted mortality rates as well as utilization rates was observed in Hispanic veterans with clinically diagnosed TBI compared to non-Hispanic White veterans. But, in a multivariable model, the direct predictive effect of Hispanic race on mortality was reduced from (HR=2.35, 95% CI 1.5, 3.68) to non significance (HR=1.58, 95% CI 0.98, 2.54). This shows partial mediation by utilization (especially utilization of neurology services) and partial confounding by covariates.

This finding appears to support the conceptual model that a process outcome, utilization, is a partial pathway for antecedent (race/ethnicity) disparities in a clinical outcome, mortality. Further evidence to support this result is our findings that Hispanic race/ethnicity is associated with a lower likelihood of having TBI clinic, neurology, rehabilitation and other types of visits.

Our findings are consistent with previous studies that have found racial/ethnic disparities in outcomes following TBI. (Arango-Lasprilla, & Kreutzer, 2011) Possible factors that have been found to be important in racial/ethnic disparities in other diseases include: socioeconomic status, (Whitfield, Weidner, Clark, & Anderson, 2003; Smart & Smart, 1992) quality of education, spirituality (Simoni & Ortiz, 2003), motivation for rehabilitation, access to care (Prieto, McNeil, Walls, & Gomez, 2001) quality of care, transportation, locus of control, social support, discrminination (Whitfield, Weidner, Clark, & Anderson, 2003; Clark, Anderson, Clark, & Williams, 1999), mistrust (Whaley, 2002), familismo, acculturation (Prieto, McNeil, Walls, & Gomez, 2001; Marin & Marin, 1991; Lara, Gamboa, Kharmanian, Morales, & Bautista, 2005; Bethel & Schenker, 2005), primary language and immigration status (Whitfield, Weidner, Clark, & Anderson, 2003).

Our findings are also consistent with the Coyle & Battles model, confirming how a process variable (utilization) can be a partial pathway between disparities in an antecedent (race/ethnicity) and an outcome (mortality). (Coyle & Battles, 1999)

The role of differential pattern of utilization as a partial pathway for ethnic differences in mortality has not been studied in the TBI population, so this study provides new evidence on the importance of ensuring equal access to key TBI related services for individuals with TBI. We show evidence that neurology appears to be most critical but it will be important to validate these findings in other populations and longitudinally so that appropriate interventions can be developed to increase utilization of critical TBI related services. Previous literature from VA and non-VA settings have made recommendations to reduce ethnic disparities that may be relevant to increasing utilization of TBI related services for veterans with TBI (Whitfield, Weidner, Clark, & Anderson, 2003; Saha, Freeman, Toure, Tippens, Weeks, & Ibrahim, 2008). The use of decision aids and information tools, patient activation interventions, and adherence support interventions (Saha, Freeman, Toure, Weeks, & Ibrahim, 2008) will be valuable in improving utilization of TBI related services, especially ethnic minorities.

Our study has a number of limitations. The cohort is for one year of veterans clinically diagnosed with TBI, though this cohort was followed for four years for mortality. We only included clinically diagnosed TBI, a group that is different from those screened via the VA primary screen. Our data did not allow us to ascertain the cause of death and we did not have data on potentially important factors associated with mortality such as time between injury and diagnosis, branch of military, Department of Defense or civilian treatment, severity, Glasgow Coma Score, discharge disposition, education and family support. We also had a high number of missing race/ethnicity values which we controlled for by including missing race/ethnicity as a separate category. Finally, we do not include non VA health care utilization which could be significant, especially for veterans eligible for Medicare either by age or by permanent disability status. However, the literature has found that dual insurance coverage does not improve mortality for veterans with another neurological disorder, stroke, so we do not see this limitation as invalidating our results. (Jia et al., 2007)

In conclusion, we sought to test a primary hypothesis that utilization would partially explain the significant racial/ethnic differential in mortality which has been found among veterans with TBI. We also tested a secondary hypothesis that race/ethnicity will be associated with lower utilization of specificTBI related services. We found that Hispanic ethnicity was associated with higher mortality among veterans with TBI and utilization, especially neurology, is a significant partial mediator in mortality. In addition, we found that Hispanic ethnicity was associated with lower utilization of TBI specific services: TBI clinic, neurology, rehabilitation and all types of services. Innovative approaches to improving utilization of TBI related services should be a priority for the VA, given the “signature wound” status of TBI in the OEF/OIF/OND veterans returning from Iraq and Afghanistan. These services may also be important in non-mortality outcomes such as quality of life, functional outcomes, employment and other vocational outcomes, and social outcomes, where race/ethnicity differences have been observed in the civilian literature (Arango-Lasprilla & Kreutzer, 2011).

**Abbreviations: traumatic brain injury (TBI)**
(VA): Veterans Affairs
(ED): emergency department
(CDC): Center for Disease Control
(VHA): Veterans Health Administration
(DSS): Decision Support System
(SSN): Social Security Number
(PTF): patient treatment file
(OEF/OIF/OND): Operation Enduring Freedom/Operation Iraqi Freedom /Operation New Dawn
(RUG): Research User Guide
(HR): hazard ratios
